# Economic Evaluations of Algorithm-Enabled Remote Monitoring of Adults With Cardiac Implantable Electronic Devices: Protocol for a Systematic Review

**DOI:** 10.2196/89974

**Published:** 2026-04-29

**Authors:** Hanan Daghash, Ryan Kenny, Cen Cong, Rohan Pandey, Edward Meinert, Gurdeep S Sagoo

**Affiliations:** 1 Translational and Clinical Research Institute, Newcastle University, Newcastle upon Tyne, England, United Kingdom; 2 Population Health Sciences Institute, Faculty of Medical Sciences, Newcastle University, Newcastle upon Tyne, England, United Kingdom; 3 Evidence Synthesis Group, Population Health Sciences Institute, Newcastle University, Newcastle upon Tyne, England, United Kingdom; 4 Department of Primary Care and Public Health, School of Public Health, Imperial College London, Exhibition Rd, South Kensington, London, England, SW7 2AZ, United Kingdom, +44 20 7589 5111

**Keywords:** systematic review protocol, economic evaluation, cost-effectiveness, cardiac implantable electronic device, CIED, remote monitoring

## Abstract

**Background:**

Cardiac implantable electronic devices (CIEDs) are crucial in managing various cardiac conditions, but their monitoring poses considerable challenges. Algorithm-enabled remote monitoring of these devices has emerged as a promising solution to enhance patient outcomes and potentially reduce health care expenditures; however, its economic impact remains underexplored.

**Objective:**

This systematic review protocol aims to review and synthesize the existing evidence on the cost-effectiveness and cost-utility of algorithm-enabled remote monitoring for CIEDs in patients with or at risk of heart failure.

**Methods:**

The search of literature will be performed in MEDLINE, Embase, Scopus, Web of Science, and the Cochrane Library, with supplementary searches in the National Health Service Economic Evaluation Database, the National Institute for Health and Care Excellence, the Canadian Agency for Drugs and Technologies in Health, the International Network of Agencies for Health Technology Assessment, and the National Institute for Health and Care Research. This protocol is reported in accordance with the PRISMA-P (Preferred Reporting Items for Systematic Reviews and Meta-Analysis Protocols) 2015 statement, and the completed review will be reported following the PRISMA 2020 statement.

**Results:**

Following database searching and deduplication, 3108 records were retrieved; 731 (23.5%) duplicates were removed, leaving 2377 (76.5%) records for title and abstract screening. The review will identify and synthesize economic evaluations of algorithm-enabled remote monitoring in adults with CIEDs, summarizing reported costs, outcomes, and cost-effectiveness results. Methodological quality, risk of bias, and sources of heterogeneity across studies will be assessed.

**Conclusions:**

The findings of this review may help inform health care providers, policymakers, and other stakeholders by clarifying the current economic evidence on these monitoring systems, informing adoption decisions, and identifying areas requiring further research.

## Introduction

### Background

Heart failure (HF) is a progressive syndrome characterized by ventricular dysfunction leading to dyspnea, fatigue, and reduced exercise capacity [1]. It affects approximately 1% to 2% of adults in high-income nations, increasing to over 10% among those aged above 70 years, and contributes to nearly 2% of total health care spending [2]. In the United Kingdom, HF accounts for 1% to 2% of National Health Service expenditure, and hospitalizations account for 60% to 70% of HF costs [3]. Contemporary European and UK audits report shorter hospital stays than older estimates, reflecting service changes and specialist input [4,5]. Despite therapeutic advances, prognosis after an HF admission remains poor, with 39% all-cause mortality at 1 year after discharge and 9% in-hospital mortality [5]. Current guidance emphasizes early detection of decompensation, treatment adjustment, and structured multidisciplinary follow-up to prevent readmissions and adverse events. Within this framework, remote monitoring (RM) through cardiac implantable electronic devices (CIEDs), including implantable cardioverter-defibrillators (ICDs), cardiac resynchronization therapy pacemakers (CRT-Ps), and cardiac resynchronization therapy defibrillators (CRT-Ds), has emerged as a promising complement to conventional care. RM technologies enable continuous physiological data transmission, allowing clinicians to detect early signs of deterioration [1]. An emerging form, algorithm-supported RM, uses automated data analysis and alert generation to flag possible clinical deterioration, device problems, or HF decompensation. In this review, algorithm-enabled RM refers to CIED RM that uses an HF algorithm to analyze and collate clinical data recorded by the device, detect gradual worsening of HF, and send alerts to health care professionals to prompt review. However, although RM shows potential for reducing in-person visits and improving workflow, its long-term clinical and economic value remains uncertain.

Evidence for the clinical and service impact of CIED-RM is mixed and context dependent. In a multicenter randomized trial, RM did not reduce all-cause mortality or cardiovascular hospitalization compared with standard care but accelerated clinical decision-making and reduced scheduled follow-ups [6]. Conversely, a multicenter pre-post study using the HeartLogic algorithm observed fewer HF hospitalizations and shorter stays after activation [7]. Large real-world data also suggest that RM is associated with lower risks of death and cardiovascular hospitalization, fewer outpatient visits and inpatient days, and overall cost reductions driven by fewer admissions [8]. Patients who consistently use RM show better long-term outcomes than those who are less adherent [9]. However, studies using different monitoring algorithms report variable alert frequency and care responses, leading to inconsistent outcomes depending on device type and program design [10,11].

Economic evaluations of CIED-RM have primarily used cost-effectiveness analysis (CEA) and cost-utility analysis (CUA) frameworks. A randomized trial reported modest quality-adjusted life year (QALY) gains and lower patient costs from reduced clinic visits but no short-term system savings [12]. A model-based lifetime CUA estimated a favorable incremental cost-effectiveness ratio (ICER) [13]. Similarly, cost-saving estimates for CIED-RM in Germany and the Netherlands are sensitive to reimbursement assumptions [14]. Country-specific CEAs in Italy and the United Kingdom reported cost-neutral or cost-saving results [15,16], while a French modeling study confirmed cost-effectiveness under probabilistic analysis [17]. A Canadian population-based CEA found reduced mortality and hospitalizations, translating into health system savings [8]. Overall, CIED-RM is frequently reported as cost-effective, particularly when evaluated over longer time horizons or from patient perspectives; however, heterogeneity in study design, perspective, and costing methods limits direct comparability.

Taken together, the evidence indicates that CIED-RM most consistently reduces routine clinic visits and shortens time to clinical response. Effects on mortality and hospitalization remain inconsistent across randomized trials but are more favorable in observational studies [6-8,18]. Economic analyses generally report CIED-RM as cost-effective, although findings vary by perspective, time horizon, and reimbursement structure [12-14,16,17]. Methodological diversity remains substantial, ranging from short-term trial-based analyses to long-horizon model projections with stronger structural assumptions. Key factors such as false-positive alert handling, staffing and implementation costs, adherence behavior, and cross-system transferability are rarely modeled explicitly [19]. Consequently, decision-makers still lack a consistent, comparable understanding of whether CIED-RM delivers economic value, under which assumptions, and for which patient groups.

Most existing reviews of CIED-RM focus on clinical outcomes and show mixed effects without a detailed economic appraisal [18]. Raes et al [20] reviewed economic evaluations of CIED telemonitoring without distinguishing between algorithm-enabled systems and basic scheduled-transmission follow-up. Their review did not describe algorithm-supported platforms, examine alert performance or handling, or assess the structural and parameter assumptions underpinning model-based economic results [20]. A National Institute for Health and Care Research (NIHR) Health Technology Assessment (HTA) report conducted a systematic review of economic evaluations for selected algorithms, providing valuable insights, but was restricted to manufacturer-specific submissions [19]. Unlike these previous reviews, this study focuses specifically on algorithm-enabled RM for patients with HF or at risk of developing HF, examining how model design, analytic perspective, time horizon, and approaches to uncertainty influence cost-effectiveness conclusions.

### Objectives

This review will systematically identify, appraise, and synthesize full economic evaluations of algorithm-enabled RM for CIEDs to describe the economic modeling structures, assumptions, and analytic perspectives used; summarize and compare the key cost components and outcome measures reported across studies; and identify methodological gaps that limit model development, comparability, and policy decision-making.

## Methods

### Overview

This protocol was developed in accordance with the PRISMA-P (Preferred Reporting Items for Systematic Review and Meta-Analysis Protocols) 2015 statement [21,22] to ensure transparency and reproducibility (Checklist 1). The review will follow methodological guidance from the Cochrane Handbook for Systematic Reviews of Interventions [23]. The protocol is prospectively registered in PROSPERO (CRD420251179324).

### Eligibility Criteria

The review’s eligibility parameters are structured using the population, intervention, comparator, outcomes, and study design (PICOS) framework.

#### Population

Adult participants aged 18 years and older diagnosed with HF or at risk of HF and treated with implantable cardiac devices (ICD, CRT-P/CRT-D, or pacemaker) will be included. No upper age limit will be applied. Participants described in the primary studies as being at risk of HF will be considered eligible according to the definitions used by the original study authors. Where reported, relevant clinical characteristics, such as New York Heart Association class, will be extracted.

#### Interventions and Comparators

The interventions of interest are algorithm-enabled RM systems integrated with implantable cardiac devices for monitoring HF status and detecting worsening. Eligible interventions include RM that uses an HF algorithm and generates alerts for clinical review across ICDs, CRT-Ps, CRT-Ds, and pacemakers, regardless of manufacturer. Comparators will include standard in-person follow-up, usual care without RM, or alternative RM strategies.

#### Costs and Outcome Measures

Eligible studies will report at least 1 economic outcome, including ICER, incremental cost per QALY gained, incremental cost per disability-adjusted life year, cost-benefit ratio, or net monetary benefit.

Clinical outcomes specific to HF, such as hospitalizations, mortality, and device-related adverse events, and outcomes related to false-positive and false-negative alerts will also be extracted.

#### Study Design

This review will include full economic evaluations, both trial based and model based, that compare the costs and outcomes of interventions. Eligible designs include CEA, CUA, and cost-benefit analysis.

We will exclude non-English publications, studies without device-linked remote algorithms, papers not focused on economic outcomes, methodological papers or reviews lacking primary studies, and conference abstracts with insufficient data.

### Information Sources

A comprehensive and systematic search will be conducted across the following electronic databases: MEDLINE (Ovid), Embase (Ovid), Scopus, Web of Science, and the Cochrane Library. We will also search HTA and guideline repositories such as the National Health Service Economic Evaluation Database, the National Institute for Health and Care Excellence, the Canadian Agency for Drugs and Technologies in Health, the International Network of Agencies for HTA, and the NIHR Journals Library. These databases have been selected to ensure adequate coverage of both clinical and economic literature. The reference lists of all included studies and relevant systematic reviews will be hand-searched to identify additional eligible publications not captured by the electronic searches.

### Search Strategy

The search strategy will be developed using a combination of MeSH (Medical Subject Headings) and free-text keywords, structured around the core concepts of implantable cardiac devices, RM, and economic evaluation. The terms will be informed by a scoping analysis of titles, abstracts, and keywords from relevant studies.

### Data Management

All retrieved references will be exported into EndNote (version 21; Clarivate) for citation management and duplicate removal. Following deduplication, the records will be imported into Rayyan (Rayyan Systems Inc) for study selection.

### Selection Process

We will use a 2-stage selection procedure. One reviewer (HD) will screen all records against the prespecified criteria. A second reviewer (RP) will independently verify a random 20% sample to assess concordance. This approach was adopted due to limited time and resource constraints and represents a pragmatic strategy to support feasibility, although it may carry a greater risk of missed studies or errors than full independent duplicate screening.

Full-text screening will be undertaken by HD, with RP independently verifying a random 20% sample to assess consistency. For excluded papers, a single primary reason will be recorded using a standardized list. Disagreements will be resolved by consensus; if unresolved, a third reviewer will decide.

Screening will be conducted using Rayyan, with blinding enabled during the verification stage. The study selection process will be documented using a PRISMA (Preferred Reporting Items for Systematic Review and Meta-Analyses) 2020 flow diagram.

### Data Extraction

We will develop a structured data extraction form in Excel (version 16.86; Microsoft). HD will extract data from all included studies, and RP will independently check a random 20% sample to assess accuracy and consistency. This approach was also adopted due to limited time and resource constraints and is intended to support feasibility. Any discrepancies will be resolved through discussion, with involvement of a third reviewer if needed.

Extracted items will include study identifiers (author, year, title, type of publication [eg, abstract, full text, and HTA report], country, setting, and funder); study design (trial based or model based); population characteristics; and details of the intervention and comparator, including the RM algorithm, device type (eg, ICD, CRT-P, CRT-D, and pacemaker), monitored parameters, alert frequency, and clinical response workflow. Economic evaluation data will cover the type of analysis, perspective, time horizon, discount rate, modeling approach, costs, price year, and currency. Outcomes will include clinical composite end points commonly reported in RM studies (such as combinations of HF hospitalizations and related events) and economic results (such as hospitalizations avoided, QALYs, and ICERs), alert performance metrics (sensitivity, specificity, positive predictive value, and false-positive rate), and sensitivity analyses. Where reported, implementation-related factors, such as workflow-related elements and alert-processing considerations, will be extracted and described narratively in the synthesis.

### Risk of Bias and Methodological Quality Assessment

HD will assess the risk of bias for all included studies, and RP will independently verify a random 20% sample to check accuracy and consistency. For randomized trials, risk of bias will be assessed using the Risk-of-Bias tool (version 2; Cochrane; randomization process, deviations from intended interventions, missing outcome data, outcome measurement, and selection of the reported result) [24]. For nonrandomized comparative studies, risk of bias will be assessed using the ROBINS-I tool (version 1; Cochrane; confounding, selection of participants, classification of interventions, deviations from intended interventions, missing data, outcome measurement, and selection of the reported result) [25]. Methodological quality of full economic evaluations will be assessed using the Drummond and Jefferson checklist [26]. For model-based economic evaluations, we will additionally apply the Philips checklist to appraise model structure, data inputs, validation, and uncertainty [27]. Reporting quality will be assessed using the Consolidated Health Economic Evaluation Reporting Standards (CHEERS) statement [28]. These assessments will inform synthesis by stratifying findings by study quality or risk of bias and by highlighting conclusions that rely on studies with major methodological limitations.

The overall certainty of evidence will be summarized qualitatively, considering study design, risk of bias, consistency, precision, and applicability. This assessment will draw on principles adapted from the GRADE (Grading of Recommendations Assessment, Development, and Evaluation) Evidence-to-Decision framework for economic evaluations (GRADE Guidance 23), focusing on model structure, data sources, uncertainty, and relevance to decision-making contexts [29]. Although GRADE is not formally designed to rate the certainty of economic model evidence, its conceptual domains will be used to guide transparent judgment of confidence in the body of evidence across included studies.

### Data Synthesis

Data will be synthesized narratively following guidance for narrative synthesis and synthesis without meta-analysis [30]. We will use structured descriptions, summary statistics, and comparative tables to highlight study characteristics, modeling approaches, cost and outcome measures, and key determinants of cost-effectiveness. Particular attention will be given to methodological differences such as model structure, perspective, time horizon, parameter sources, and the treatment of false-positive and false-negative alerts as potential drivers of variation in incremental cost-effectiveness results.

Given the expected heterogeneity in study designs, health care systems, and modeling assumptions, a quantitative meta-analysis may not be appropriate. However, if at least 3 studies report comparable cost-effectiveness outcomes with available variance data, a random-effects model will be explored. Comparable outcomes will require the same perspective, time horizon, outcome metric (eg, ICER and QALY), and a common (or convertible) currency and price year. All findings will be presented using summary tables and descriptive text and reported in accordance with the PRISMA 2020 statement [21].

## Results

**Figure 1.**
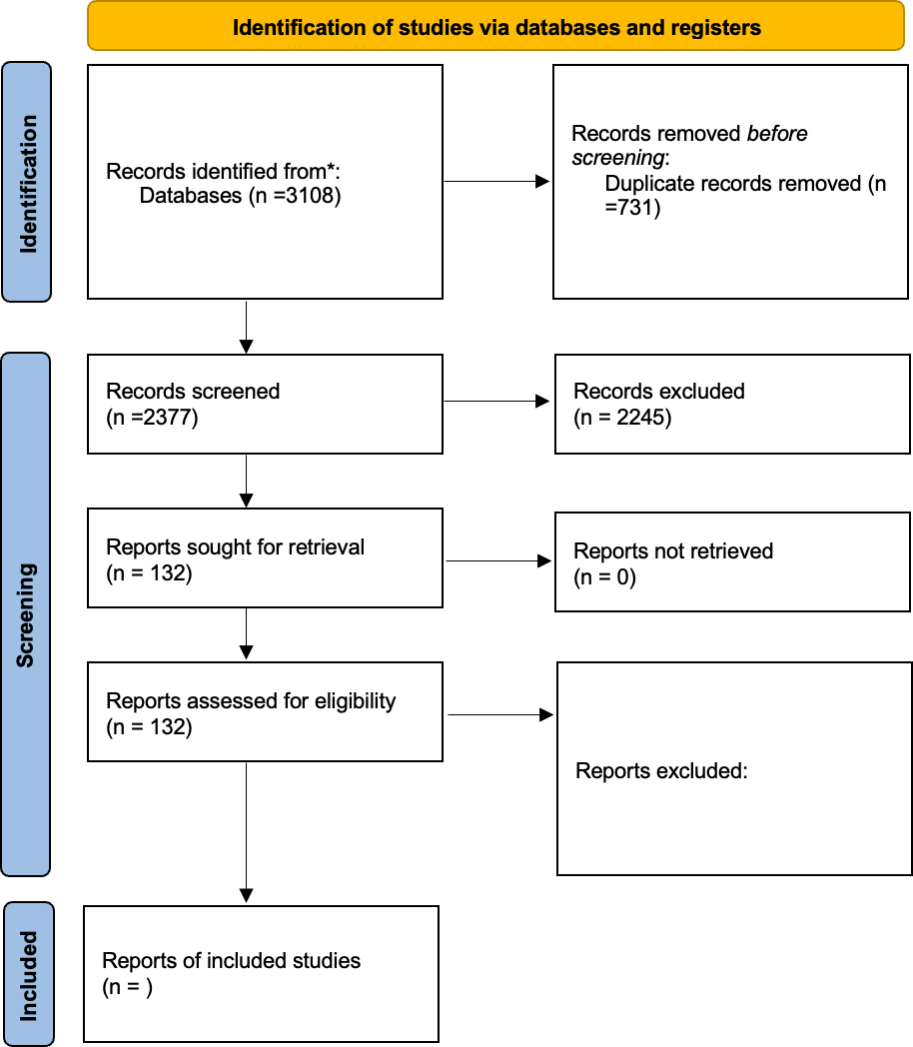
PRISMA (Preferred Reporting Items for Systematic Reviews and Meta-Analyses) flow diagram of the study selection process.

Database and supplementary searches identified 3108 records. After removal of 731 (%) duplicates, 2377 (%) records remained for title and abstract screening. Following title and abstract screening, 132 (%) full-text papers were assessed for eligibility (Figure 1). The completed review is expected to identify and synthesize full economic evaluations of algorithm-enabled RM in adults with CIEDs, summarizing economic outcomes, clinical outcomes (including patient-reported outcomes), and cost-effectiveness results. It is also expected to describe modeling approaches and highlight methodological gaps relevant to future model development.

## Discussion

### Anticipated Findings

This systematic review is expected to identify the range of economic analyses used in evaluations of algorithm-enabled RM for CIEDs, with particular attention to analytic perspective, time horizon, and sensitivity analyses. It is also expected to clarify the assumptions, data sources, and modeling approaches used in current health economic evaluations. In addition, the review is expected to describe the clinical and economic outcomes reported across studies, as well as how the algorithms function, which parameters they use, and how alerts are linked to clinical response pathways. By applying tools for risk of bias, methodological quality, and reporting quality, the review is also expected to highlight the strengths, limitations, and reporting gaps in the current evidence base.

This systematic review differs from previous reviews by focusing specifically on the economic evaluation of algorithm-enabled RM for patients with CIEDs who have HF or are at risk of developing HF. A previous review by Raes et al [20] examined telemonitoring in patients with CIEDs more broadly and did not focus specifically on HF algorithms or on patients with HF or at risk of HF. CIED-based monitoring can be used for several purposes, including detection of arrhythmias, atrial fibrillation, and HF deterioration, whereas this review is restricted to algorithm-enabled RM systems designed to detect worsening HF or risk of decompensation. In addition, a recent NIHR HTA provided valuable evidence on selected manufacturer-submitted technologies; however, its scope was limited to 4 specific algorithms [19]. In contrast, this review aims to provide a broader synthesis of published economic evaluations of algorithm-enabled RM in CIED populations with HF or at risk of HF.

This review has several strengths. It adopts a focused scope by examining algorithm-enabled RM in patients with HF or at risk of HF. In addition, the review extends beyond outcome reporting by examining modeling structures, assumptions, and analytical perspectives, providing methodological insights relevant to future economic evaluations. The use of multiple established appraisal tools, including the Drummond and Jefferson checklist, the Philips checklist, and the CHEERS statement, further strengthens the robustness of the assessment. A comprehensive search strategy across multiple databases and HTA sources also enhances the coverage of relevant studies.

However, several limitations should be considered. Screening and data extraction were primarily conducted by a single reviewer with partial verification, which may increase the risk of error compared with full duplicate review and should be considered when interpreting the findings. Substantial heterogeneity in study design, modeling approaches, and outcome measures may limit comparability and restrict quantitative synthesis. Restricting inclusion to English-language publications may introduce language bias. In addition, the generalizability of findings may be limited, as economic results are context specific and influenced by health care system characteristics. Finally, the strength of conclusions will depend on the quality and reporting of the included studies.

The findings of this review may help inform future economic evaluations, adoption decisions, and modeling studies of algorithm-enabled RM for CIEDs. The review may also highlight methodological gaps requiring further research, particularly in relation to alert management, workflow, adherence, and generalizability across health care settings. In addition, the findings from this review may help inform future patient and public involvement work to identify and prioritize outcomes that matter most to patients in the context of RM, including quality of life, burden, and anxiety related to alerts. The findings of this review will be disseminated through a peer-reviewed publication and conference presentations.

### Conclusions

This systematic review is expected to provide a clearer understanding of the current economic evidence base for algorithm-enabled RM for CIEDs. It is also expected to identify key economic drivers and highlight methodological gaps in existing models that may affect the reliability, comparability, and decision relevance of the available evidence.

## Abbreviations

CEA: cost-effectiveness analysis
CHEERS: Consolidated Health Economic Evaluation Reporting Standards
CIED: cardiac implantable electronic device
CRT-D: cardiac resynchronization therapy defibrillator
CRT-P: cardiac resynchronization therapy pacemaker
CUA: cost-utility analysis
GRADE: Grading of Recommendations Assessment, Development, and Evaluation
HF: heart failure
HTA: Health Technology Assessment
ICD: implantable cardioverter-defibrillator
ICER: incremental cost-effectiveness ratio
MeSH: Medical Subject Headings
NIHR: National Institute for Health and Care Research
PICOS: population, intervention, comparator, outcomes, and study design
PRISMA: Preferred Reporting Items for Systematic Review and Meta-Analyses
PRISMA-P: Preferred Reporting Items for Systematic Review and Meta-Analysis Protocols
QALY: quality-adjusted life year
RM: remote monitoring
